# EfficientNet-Based System for Detecting EGFR-Mutant Status and Predicting Prognosis of Tyrosine Kinase Inhibitors in Patients with NSCLC

**DOI:** 10.1007/s10278-024-01022-z

**Published:** 2024-02-15

**Authors:** Nan Xu, Jiajun Wang, Gang Dai, Tao Lu, Shu Li, Kexue Deng, Jiangdian Song

**Affiliations:** 1 School of Health Management, China Medical University, Shenyang, Liaoning, 110122 China; 2 Department of Thoracic Surgery, The First Affiliated Hospital of China Medical University, Shenyang, Liaoning, 110001 China; 3 Department of Radiology, The First Affiliated Hospital of University of Science and Technology of China (USTC), Division of Life Sciences and Medicine, USTC, Hefei, Anhui 230036 China; 4 Department of Radiology, The First Affiliated Hospital of China Medical University, Shenyang, Liaoning, 110001 China

**Keywords:** Artificial intelligence, Non-small cell lung cancer, Epidermal growth factor receptor, Prognosis

## Abstract

We aimed to develop and validate a deep learning-based system using pre-therapy computed tomography (CT) images to detect epidermal growth factor receptor (EGFR)-mutant status in patients with non-small cell lung cancer (NSCLC) and predict the prognosis of advanced-stage patients with EGFR mutations treated with EGFR tyrosine kinase inhibitors (TKI). This retrospective, multicenter study included 485 patients with NSCLC from four hospitals. Of them, 339 patients from three centers were included in the training dataset to develop an EfficientNetV2-L-based model (EME) for predicting EGFR-mutant status, and the remaining patients were assigned to an independent test dataset. EME semantic features were extracted to construct an EME-prognostic model to stratify the prognosis of EGFR-mutant NSCLC patients receiving EGFR-TKI. A comparison of EME and radiomics was conducted. Additionally, we included patients from The Cancer Genome Atlas lung adenocarcinoma dataset with both CT images and RNA sequencing data to explore the biological associations between EME score and EGFR-related biological processes. EME obtained an area under the curve (AUC) of 0.907 (95% CI 0.840–0.926) on the test dataset, superior to the radiomics model (*P* = 0.007). The EME and radiomics fusion model showed better (AUC, 0.941) but not significantly increased performance (*P* = 0.895) compared with EME. In prognostic stratification, the EME-prognostic model achieved the best performance (C-index, 0.711). Moreover, the EME-prognostic score showed strong associations with biological pathways related to EGFR expression and EGFR-TKI efficacy. EME demonstrated a non-invasive and biologically interpretable approach to predict EGFR status, stratify survival prognosis, and correlate biological pathways in patients with NSCLC.

**Supplementary Information:**

The online version contains supplementary material available at 10.1007/s10278-024-01022-z.

## Introduction

In terms of morbidity and mortality, lung cancer is one of the most common cancers worldwide [1]. Non-small cell lung cancer (NSCLC) has been shown to account for approximately 80–85% of all lung cancer cases [2]. Identifying activating mutations in the epidermal growth factor receptor (EGFR) in patients with NSCLC and developing targeted therapies with EGFR tyrosine kinase inhibitors (TKI) have drastically improved the clinical diagnosis and treatment of NSCLC. Currently, the detection of EGFR mutations relies mainly on invasive tumor tissue testing. However, approximately 75% of patients with NSCLC have advanced-stage tumors at diagnosis, making surgical resection or pathological puncture impractical [3]. Moreover, EGFR gene sequencing faces significant limitations, such as high genetic heterogeneity of NSCLC and poor DNA quality [4]. On the other hand, previous studies have reported that the response rate to clinical EGFR-TKI therapies in EGFR-mutant NSCLC patients is approximately 70%, whereas the remaining 30% of patients with positive EGFR mutations do not show the expected survival outcomes [5–7]. In addition, clinical studies have indicated that stratifying the prognosis of patients based solely on clinical characteristics such as gender and smoking history was not satisfactory [8, 9]. Therefore, developing a non-invasive method to detect the EGFR-mutant status and predict whether patients could benefit from EGFR-TKI is critical.

Tremendous progress has been made using artificial intelligence, especially deep learning, in the clinical diagnosis and treatment of NSCLC [10–13]. Previous studies have revealed radiogenomic correlations between gray-level texture features on intratumoral computed tomography (CT) and EGFR mutation status in patients with lung cancer [8, 11, 14, 15]. Meanwhile, it has been proven that the morphometric features of medical images, which are imperceptible to human eyes but detectable by deep learning, are associated with the prognosis of patients with NSCLC [13, 16, 17]. Most previous studies have proposed deep learning models that achieve only a single goal, and rarely achieve both EGFR gene mutation status detection and prognostic evaluation of patients receiving EGFR-TKI therapies [15, 16]. In addition, the accuracy of the models proposed in previous studies is limited, and deep learning models with higher accuracy are needed for clinical applications [8, 15]. Moreover, current deep learning models are limited by the intrinsic “black box” attribute, and studies on the validation of the credibility and biological interpretation of these models are limited [18–20].

The EfficientNet network proposed by the Google Brain team in 2019 ensures the model’s accuracy while reducing its parameter scale [21]. The EfficientNetV2 network was proposed in 2021 as an update to the EfficientNet network, which has made remarkable achievements in the diagnosis and prognosis of various human diseases [16, 22–24].

In this study, we proposed an EfficientNetV2-L-based deep learning system (EME) to non-invasively predict the EGFR-mutant status in patients with NSCLC and stratify the prognosis of EGFR-mutant patients receiving TKI therapies. Additionally, we investigated the EME as a biologically validated approach to detect EGFR mutation and predict EGFR-TKI survival prognosis.

## Methods

### Study Design and Participants

**Fig. 1:**
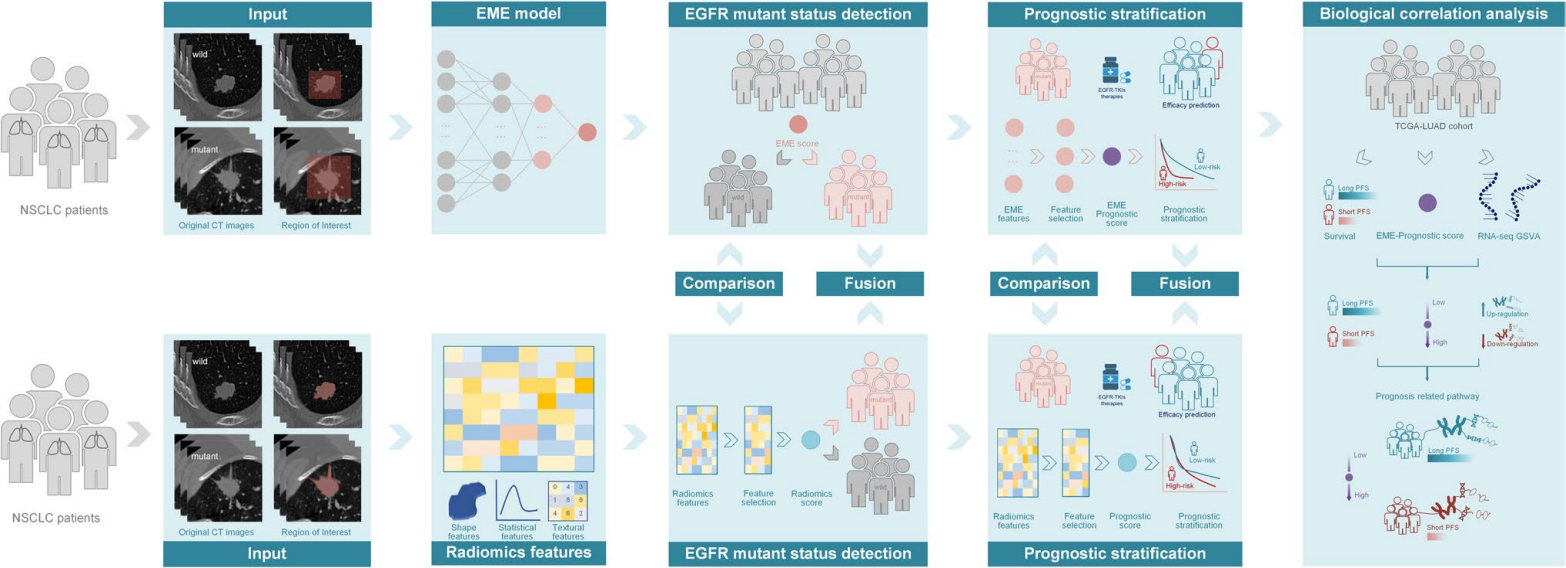
Study flowchart. CT, computed tomography; EGFR, epidermal growth factor receptor; EME, the deep learning model proposed in this study; GSVA, gene set variation analysis; NSCLC, non-small cell lung cancer; TCGA-LUAD, The Cancer Genome Atlas lung adenocarcinoma; TKIs, tyrosine kinase inhibitors; PFS, progression-free survival

This was a retrospective, multicenter study conducted in compliance with the Declaration of Helsinki and approved by the ethics committee of each center. The requirement for informed consent was waived because of the study’s retrospective nature. The overall study design is shown in Fig. 1. The inclusion criteria were as follows: (1) patients diagnosed with NSCLC in four centers from north, eastern, and central China between January 2015 and May 2021; (2) patients who underwent CT examination before treatment; (3) patients who underwent an EGFR-mutant status test; and (4) patients with EGFR positive mutation who were treated with the recommended EGFR-TKI therapies. The exclusion criteria were as follows: (1) patients without baseline CT images; (2) patients without records of the EGFR mutation status; and (3) patients without clinical characteristics. To reveal the correlation between EME and biological pathways of EGFR, we used patients from The Cancer Genome Atlas lung adenocarcinoma (TCGA-LUAD) dataset comprising different races from the USA and Canada (inclusion and exclusion criteria of patients from the TCGA-LUAD dataset are presented in *Supplementary *A). In this study, patients from the first three hospitals were used as the training dataset, and those from the remaining hospitals were the test dataset. The number of patients in the training and test datasets to develop and validate the EME model follows the 70%:30% principle.

According to the EGFR mutation testing (for detailed procedures, see *Supplementary *B), patients were labeled as EGFR-mutant or EGFR wild-type. Patients could have multiple EGFR mutation types, such as exon 19 deletions and L858R mutations [25]. Patients without any detected EGFR mutations were labeled as EGFR wild-type. For patients with EGFR mutations, follow-up of progression-free survival (PFS), defined as the time from initiation of treatment to the first occurrence of disease progression according to RECIST 1.1 criteria or death from any cause [26], was recorded.

All baseline CT images were reviewed by local radiologists. The ITK-SNAP software [27] (version 3.6.0; http://www.itksnap.org) was used to segment the regions of interest of the primary CT scans by two radiologists with > 5 years of experience. An NSCLC expert with > 10 years of experience, blinded to the above two radiologists, reviewed all the segmentation results. The detailed CT acquisition and reconstruction parameters are described in *Supplementary *C.

### Construction of the EME Model

Previous studies indicated that the peritumoral area on medical images may be highly correlated with disease progression and prognosis in patients with NSCLC [28–30]. Therefore, not only the tumor region but also, at most, 10 mm of its peripheral region was included in a patch. The patches were then resized to 256 × 256 pixels with three channels to input into the EME model.

The EME model was based on the cutting-edge EfficientNetV2-L model [31]. The details of the development of the EME model are listed in *Supplementary *D. The output of the EME is the score predicting the presence or absence of EGFR mutations for each tumor slice. For each patient, the EME score was calculated by averaging the scores for all tumor slices.

Further, to stratify the PFS, we used LASSO Cox regression to build a basic EME-prognostic model based on the deep learning features extracted from the last layer connected to the fully connected layer. A stepwise regression method based on the Akaike information criterion (AIC) was applied to construct the final EME-prognostic model [32]. Once the EME-prognostic model was constructed, a well-validated prognostic tool for signature cut-off selection in the X-tile software (version 3.6.1) [33] was used to select the optimal cut-off score to stratify patients into high- or low-risk subgroups.

### Model Comparison and Integration

To further evaluate the performance of the EME model, radiomics features of each patient were extracted using Pyradiomics (version 3.0.1) [34]. A detailed description of the radiomics features is presented in *Supplementary *E.

Radiomics models were then constructed to predict EGFR-mutant status in patients with NSCLC and to stratify the prognosis after receiving EGFR-TKI. Additionally, fusion models were built by integrating the EME and radiomics models to verify the improvement of EME performance by integrating radiomics.

### Feature Visualization

To elucidate the “black box” of the EME model, first, the gradient-weighted class activation maps (Grad-CAM) were used to produce activation maps highlighting the relevant regions activated by the EME model [35]. In addition, we used a nonlinear dimensionality reduction method called t-distributed Stochastic Neighbor Embedding (t-SNE) to reduce the feature dimensions and visualize the relationship between the EME features and EGFR mutation status [36]. Moreover, we used a heat map to describe the relationship between the EME-prognostic features and the prognosis of EGFR-mutant NSCLC patients.

### Biological Correlation Analysis

The EME was applied to patients with NSCLC from the TCGA-LUAD dataset. Pathway enrichment of the RNA-seq data of TCGA-LUAD patients was conducted, and the top-ranked key pathways activating the EGFR mutation and regulating the response of EGFR-TKI therapy in patients with NSCLC were identified. Gene set variation analysis (GSVA) was performed to obtain the patient-level GSVA score of each key pathway, and the correlation of the EME signature and the patient-level GSVA scores were evaluated to clarify the relationship of the radiologic features and the underlying biological pathway regulation.

### Statistical Analysis

All statistical analyses were conducted using the R software (Version 4.2.0). To compare baseline characteristics, the chi-square test was used for categorical variables, and the Mann–Whitney *U* test was used for continuous variables. For predicting the EGFR mutation status, we used the area under the curve (AUC), accuracy, precision, recall, and F1 score to evaluate the performance of the models. The Delong test was used to compare the AUCs of different models [37]. The bootstrap method was used to calculate 95% confidence intervals (CI). Multivariate logistic regression analysis was performed using the “glmnet” R package to construct the radiomics model.

For prognosis evaluation, LASSO Cox regression analysis was conducted using the “glmnet” R package, and survival analysis was performed using the “Survival” R package. To reduce the complexity of the prognostic model, we used the AIC stepwise regression method to construct a final effective prognostic model with minimal AIC score. Afterward, Kaplan–Meier analysis and log-rank test were used to visualize the survival curves of the two subgroups using the “survminer” R package. Harrell’s concordance index (C-index) was used to evaluate the performance of the prognostic models. Moreover, we calculated each feature’s contribution for the prognosis task by utilizing the SHapley Additive exPlanation (SHAP) values obtained from the “kernelshap” R package [38, 39].

Additionally, the analysis of t-SNE was conducted using the “Rtsne” R package, and scatter diagrams were drawn using the “ggplot2.” A heat map of the correlation among the prognostic features was drawn using the “ComplexHeatmap” R package. Pathway enrichment was performed using the “clusterProfiler” package by querying the annotated gene set database of Gene Ontology. Biological pathways with a false discovery rate were considered to be statistically significant in the enrichment analysis. Pearson's correlation coefficient was used to determine the relationship between the EME signature and key biological pathways. Statistical significance was set at *P* < 0.05.

## Results

### Patient Characteristics

**Fig. 2:**
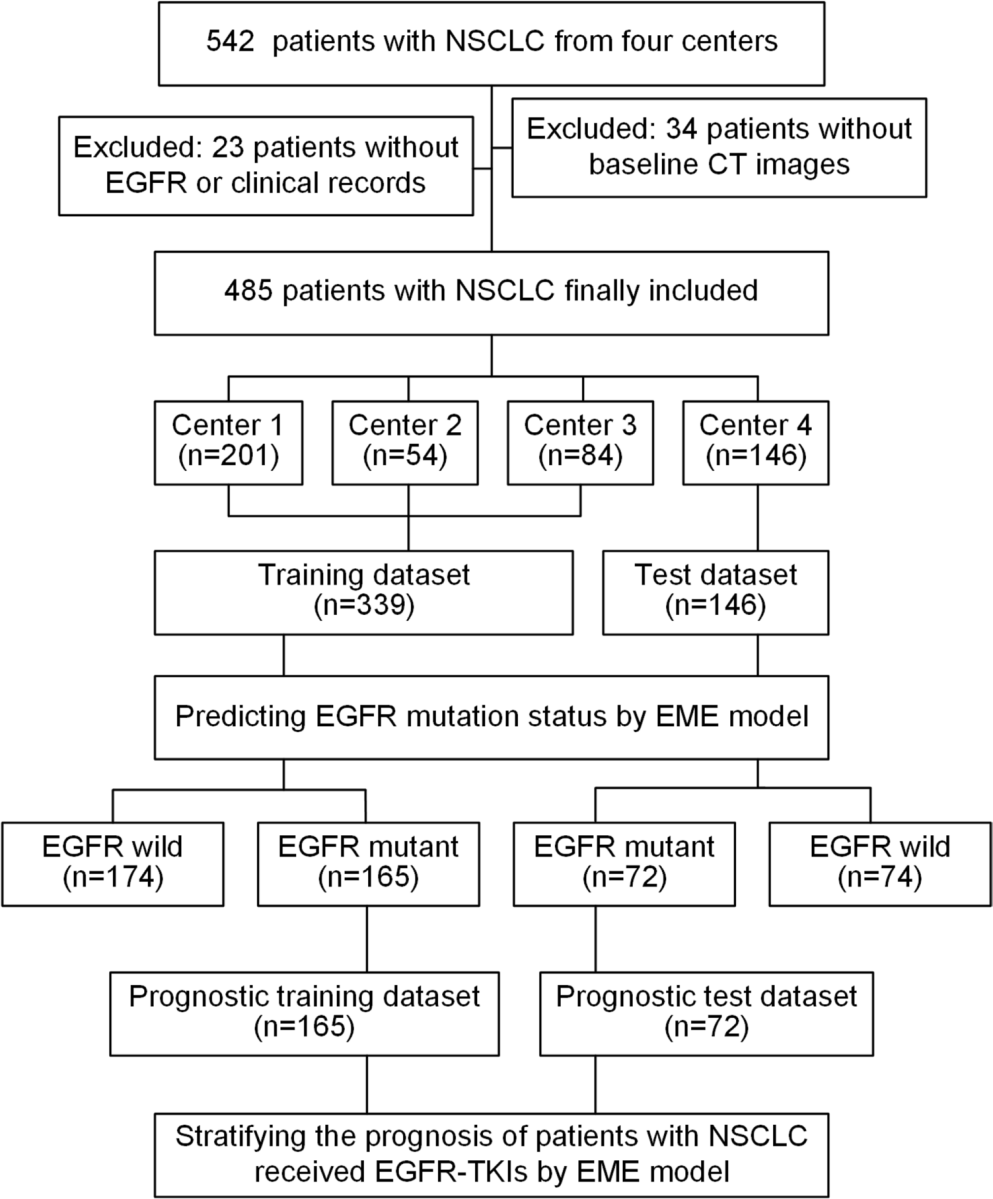
Patient enrollment. CT, computed tomography; EGFR, epidermal growth factor receptor; EME, the deep learning model proposed in this study; NSCLC, non-small cell lung cancer; TKIs, tyrosine kinase inhibitors

Between January 2015 and May 2021, 542 patients with NSCLC from four independent hospitals were recruited. As shown in Fig. 2, 485 patients with NSCLC from three centers were enrolled and grouped into a training dataset (339 patients), and the patients from the remaining fourth center were used as the test dataset (146 patients) to develop and validate the EME model. Balanced subgroups were found in our training (165 patients with EGFR positive mutation and 174 patients with EGFR wild-type) and test (72 patients with EGFR positive mutation and 74 patients with EGFR wild-type) dataset, and the detailed characteristics are presented in Table 1. The characteristics and flowchart for patients from TCGA-LUAD are presented in Supplementary Table S1 and *Fig. *S1, respectively.

**Table 1:** Baseline characteristics of all enrolled patients

	Training dataset						Test dataset		*P*
	Center 1 (*N* = 201)		Center 2 (*N* = 54)		Center 3 (*N* = 84)		Center 4 (*N* = 146)		
	EGFR (M)	EGFR (W)	EGFR (M)	EGFR (W)	EGFR (M)	EGFR (W)	EGFR (M)	EGFR (W)	
*N* (%)	111 (55.2%)	90 (44.8%)	54 (100%)	0	0	84 (100%)	72 (49.3%)	74 (50.7%)	
Age, *N* (%)									0.231
<65	47 (23.4%)	50 (24.9%)	28 (51.9%)	0	0	50 (59.5%)	40 (27.4%)	44 (30.1%)	
≥65	64 (31.8%)	40 (19.9%)	26 (48.1%)	0	0	34 (40.5%)	32 (21.9%)	30 (20.6%)	
Sex, *N* (%)									0.074
Male	47 (23.4%)	52 (25.9%)	33 (61.1%)	0	0	53 (63.1%)	44 (30.1%)	45 (30.8%)	
Female	64 (31.8%)	38 (18.9%)	21 (38.9%)	0	0	31 (36.9%)	28 (19.2%)	29 (19.9%)	
Smoke, *N* (%)									0.165
No	93 (46.3%)	49 (24.4%)	39 (72.2%)	0	0	46 (54.8%)	60 (41.1%)	28 (19.2%)	
Yes	18 (8.9%)	41 (20.4%)	15 (27.8%)	0	0	38 (45.2%)	12 (8.2%)	46 (31.5%)	
PFS, median (range)	289 (18–1948)	NA	244.5 (42–863)	NA	NA	NA	247.5 (45–1128)	NA	0.516
PFS of datasets, median (range)	269 (18–1948)						247.5 (45–1128)		

*EGFR* epidermal growth factor receptor, *N* number, *M* mutant type, *W* wild-type, *PFS* progression-free survival. PFS is measured in days

Prognostic analysis was performed for all EGFR-mutant patients (237 patients). Among them, 165 patients from 2 hospitals were included in the prognostic training dataset, with a median PFS of 269 days (range, 18–1948 days), and 72 patients from another hospital were included in the prognostic test dataset, with a median PFS of 247.5 days (range, 45–1128 days).

### Models for EGFR-Mutant Status Detection

The AUC of the EME model was 0.922 (95% CI 0.878–0.939) in the training dataset and 0.907 (95% CI 0.840–0.926) in the test dataset. The accuracy of the EME model was 0.891 (95% CI 0.866–0.915) for the training dataset and 0.829 (95% CI: 0.800–0.864) for the test dataset. There was no statistically significant difference between the training and test datasets (*P* = 0.062, Delong test). The detailed results are presented in Table 2. Notably, the EME model showed consistent performance across the three centers in the training datasets (confusion matrices in different centers are shown in *Supplementary Fig. *S2).

**Table 2:** The performance of different models in predicting EGFR-mutant status

	Accuracy	Precision	Recall	F1 score	AUC	*P*
Training dataset						
EME model	0.891 (0.866–0.915)	0.965 (0.946–0.984)	0.838 (0.802–0.868)	0.898 (0.872–0.921)	0.922 (0.878–0.939)	*Ref*
Radiomics model	0.870 (0.844–0.909)	0.820 (0.787–0.887)	0.939 (0.861–0.976)	0.876 (0.824–0.929)	0.931 (0.905–0.957)	0.538^a^
Fusion model	0.968 (0.950–0.985)	0.975 (0.941–0.981)	0.958 (0.927–0.988)	0.968 (0.935–0.982)	0.994 (0.989–0.997)	0.038^a^
Test dataset						
EME model	0.829 (0.800–0.864)	0.812 (0.772–0.847)	0.809 (0.759–0.861)	0.809 (0.774–0.837)	0.907 (0.840–0.926)	*Ref*
Radiomics model	0.801 (0.747–0.870)	0.779 (0.718–0.877)	0.833 (0.708–0.931)	0.806 (0.714–0.896)	0.825 (0.755–0.896)	0.007^b^
Fusion model	0.884 (0.849–0.938)	0.816 (0.774–0.931)	0.972 (0.847–0.986)	0.888 (0.811–0.956)	0.941 (0.905–0.978)	0.895^b^

*AUC* area under curve. EME, the deep learning model proposed in this study. The bracketed text indicates 95% confidence intervals. Ref, the EME model was used for the reference model for comparison

^a^Comparison of AUC with the EME model in the training dataset (Delong test)

^b^Comparison of AUC with the EME model in the test dataset (Delong test)

For comparison, we constructed a radiomics model containing 35 features to predict the EGFR mutation status (detailed description is provided in *Supplementary *F). The contribution and importance of the top 15 radiomics features for predicting EGFR mutation status described by SHAP values are shown in *Supplementary Fig. *S3. As shown in Table 2, the AUC of the radiomics model reached 0.825 (95% CI 0.755–0.896) in the test dataset, which is significantly lower than that of the EME model (*P* = 0.007, Delong test). Subsequently, a fusion model containing the EME score and radiomics features was constructed (a detailed description is provided in Supplementary G). The AUC of the fusion model reached 0.941 (95% CI 0.905–0.978) in the test dataset, which was significantly higher than that of the radiomics model (*P* = 0.001, Delong test). However, there was no statistically significant difference between the EME and the fusion models in the test dataset (*P* = 0.895, Delong test).

### Models for EGFR-TKI Prognostic Evaluation

The final EME-prognostic model with 49 features is presented in *Supplementary *H. The C-index of the EME-prognostic model was 0.778 (95% CI 0.732–0.825) in the prognosis training dataset and 0.711 (95% CI 0.657–0.775) in the prognosis test dataset. The contribution and importance of the top 15 EME features for survival prognosis described by SHAP values are shown in *Supplementary Fig. *S4.

**Fig. 3:**
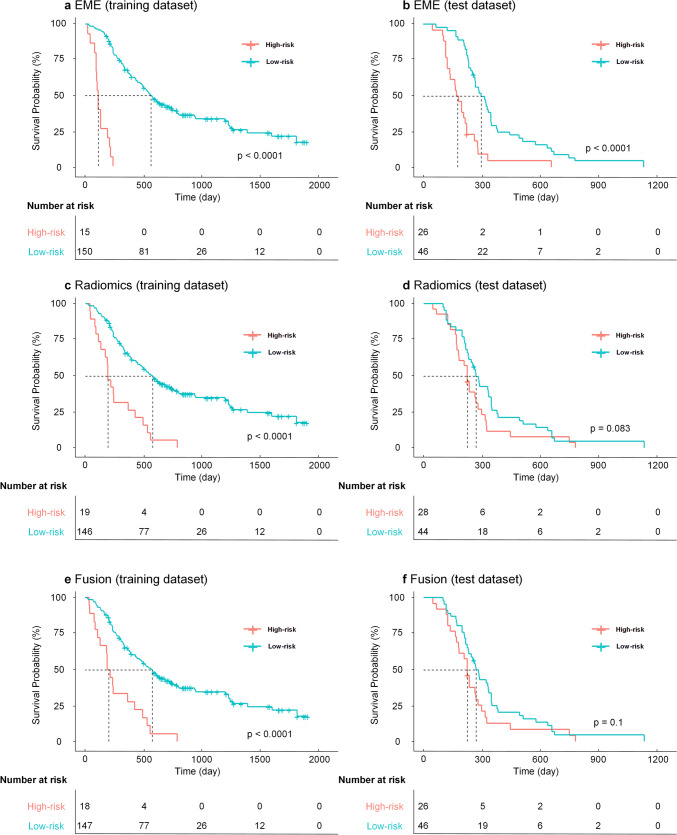
Kaplan–Meier curves of **a**, **b** the EME-prognostic model; **c**, **d** radiomics-prognostic model; **e**, **f** fusion-prognostic model in the prognostic training dataset and test dataset, respectively. EME, the deep learning model proposed in this study

X-tile indicated that an optimal cut-off of 2.16 was determined to stratify patients into high- and low-risk subgroups. The results showed that patients with an EME-prognostic score lower than 2.16 showed a significantly better PFS than those with an EME-prognostic score higher than 2.16 (training dataset, hazard ratio (HR) 0.099, 95% CI 0.022–0.453, *P* < 0.001; test dataset, HR 0.341, 95% CI 0.180–0.647, *P* < 0.001). The Kaplan–Meier curves of EGFR-mutant NSCLC patients in the prognostic training and test datasets are presented in Fig. 3.

Moreover, a radiomics-prognostic model was constructed for comparison. A total of 15 radiomics features were selected; the C-index of the prognostic model reached 0.726 (95% CI 0.678–0.773) in the prognostic training dataset and 0.551 (95% CI 0.478–0.625) in the test dataset. X-tile indicated an optimal cut-off of 1.09 on the prognosis training dataset for stratifying the risk groups (HR 0.285, 95% CI 0.127–0.643, *P* < 0.001). However, there was no statistically significant difference between the two groups in the test dataset (HR 0.691, 95% CI 0.412–1.158, *P* = 0.083). The Kaplan–Meier curves based on the three models mentioned above are shown in Fig. 3.

### Visualization

**Fig. 4:**
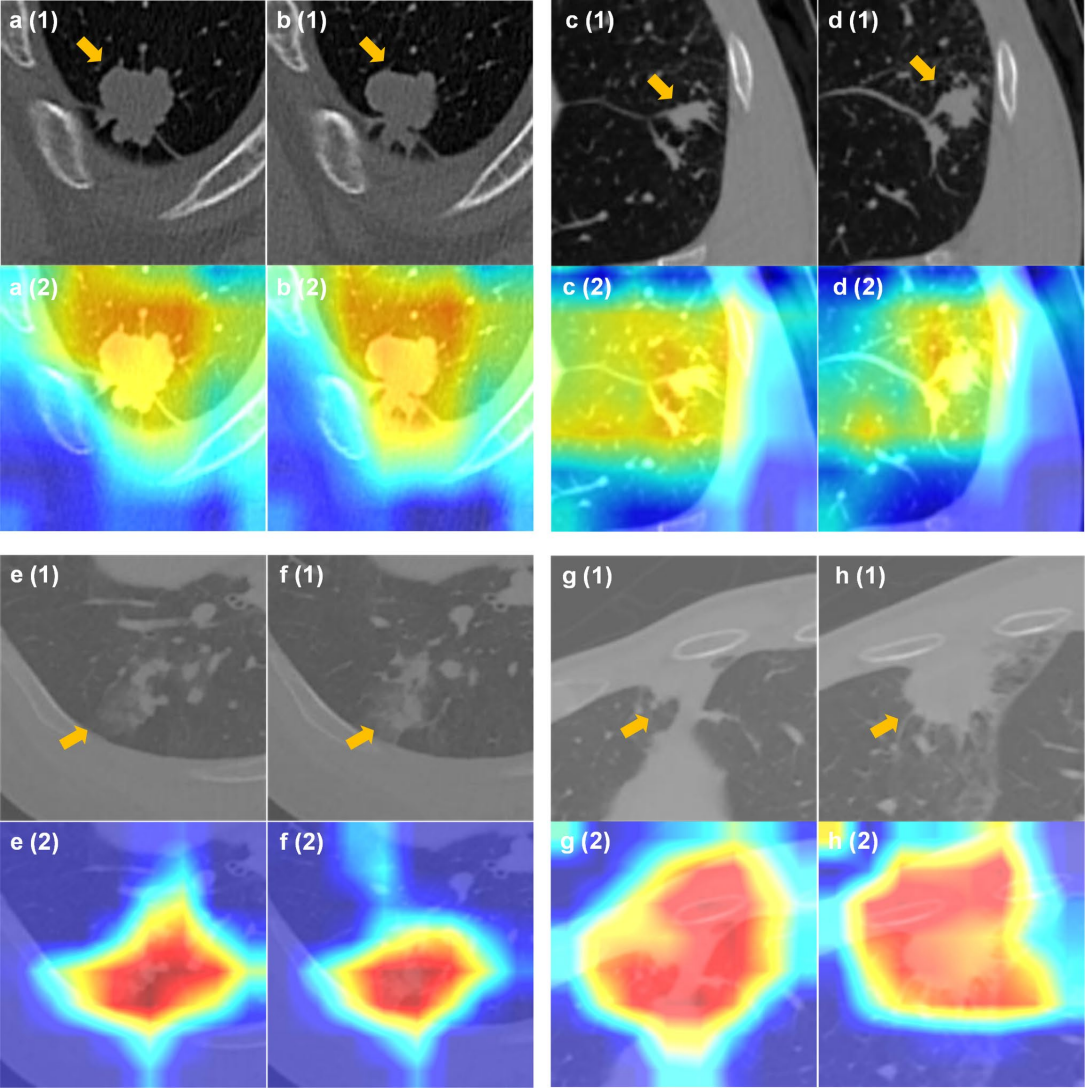
The GRAD-CAM activation maps of patients with NSCLC. (**a**-**b**) A 61-year-old male with an EME score of 0.996, biopsy-confirmed EGFR wild-type. (**c**-**d**) A 55-year-old male with an EME score of 0.996, biopsy-confirmed EGFR wild-type. (**e**–**f**) A 67-year-old male with a PFS of 630 days, biopsy-confirmed EGFR exon 19 deletion mutation (with an EME score of 0.030), and the EME-prognostic model predicted as low risk after receiving EGFR-TKI (with an EME-prognostic score of − 3.771). (**g**-**h**) A 63-year-old female with a PFS of 132 days, biopsy-confirmed EGFR exon 20 insertion mutation (with an EME score of 0.369), and the EME-prognostic model predicted as high risk after receiving EGFR-TKI (with an EME-prognostic score of 2.281). EGFR, epidermal growth factor receptor; NSCLC, non-small cell lung cancer; EGFR, epidermal growth factor receptor; PFS, progression-free survival; TKI, tyrosine kinase inhibitors

To illustrate the feature maps of EME, the GRAD-CAM activation maps of four patients with NSCLC are shown in Fig. 4, revealing that the EME model accurately targeted the locations of the intratumoral and peritumoral areas. Moreover, tumor activation maps revealed distinct imaging patterns between EGFR-mutant and EGFR wild-type patients. We found that heat maps of the EGFR-mutant patients expressed richer heterogeneity in the intratumoral and peritumoral areas, suggesting a higher probability of EGFR mutation expression than patients without EGFR mutations.

**Fig. 5:**
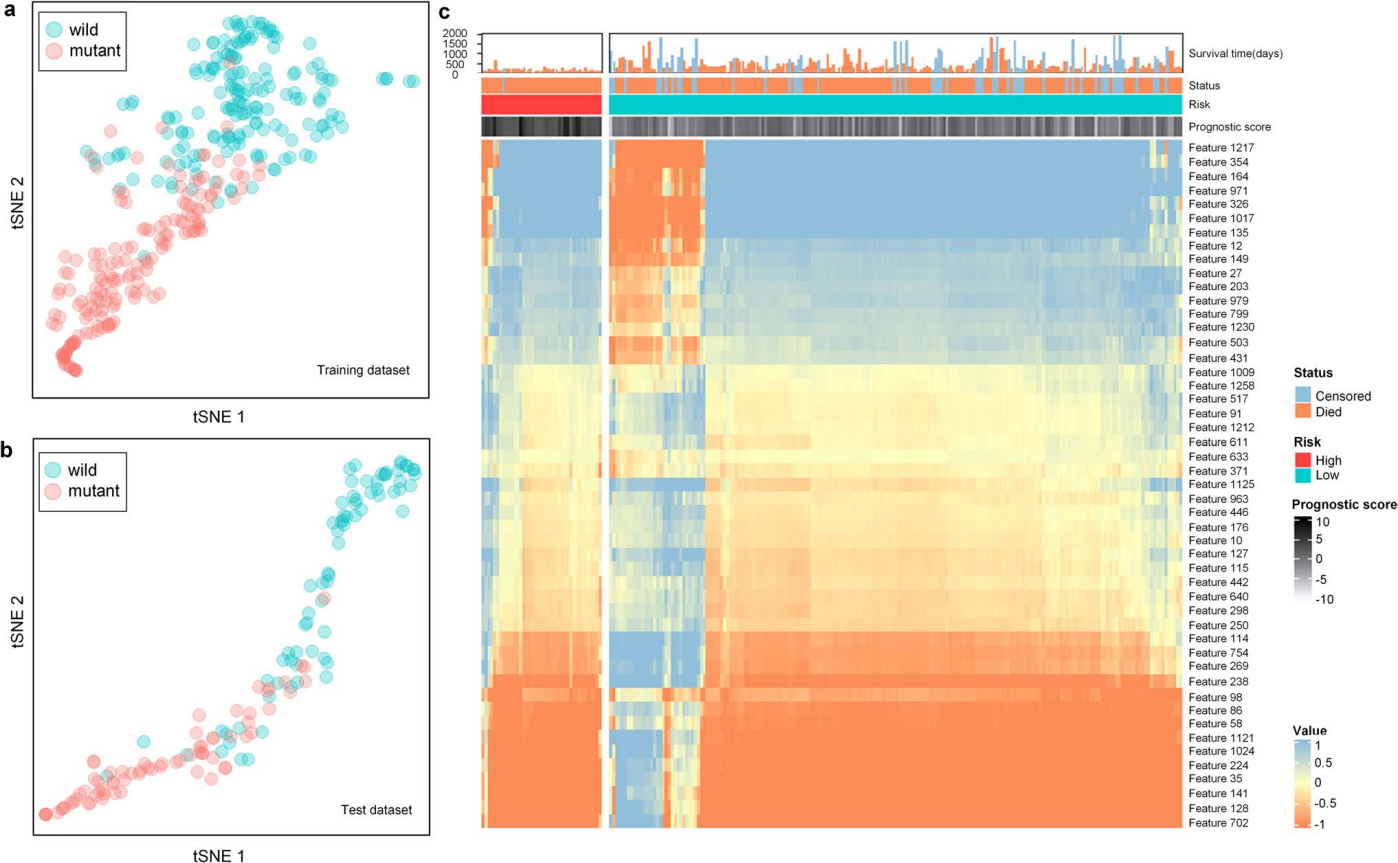
Visualization of the features in the EME system. The t-SNE analysis of the relationship between the EME features and EGFR-mutant status in the **a** training and **b** test datasets. **c** Heat map revealing the relationship between the EME-prognostic features and risk of all EGFR-mutant NSCLC patients receiving EGFR-TKI therapies. EGFR, epidermal growth factor receptor; NSCLC, non-small cell lung cancer; TKI, tyrosine kinase inhibitors; t-SNE, t-distributed Stochastic Neighbor Embedding

Further, Fig. 5a and b show the relationship between the EME features and EGFR-mutant status based on t-SNE analysis in the training and test datasets, showing that hidden features in the EME were closely related to EGFR-mutant status.

Moreover, a heat map (Fig. 5c) containing all EGFR-mutant patients showed the relationship between the EME-prognostic features and risk in patients with NSCLC.

### Biological Correlation Analysis

**Fig. 6:**
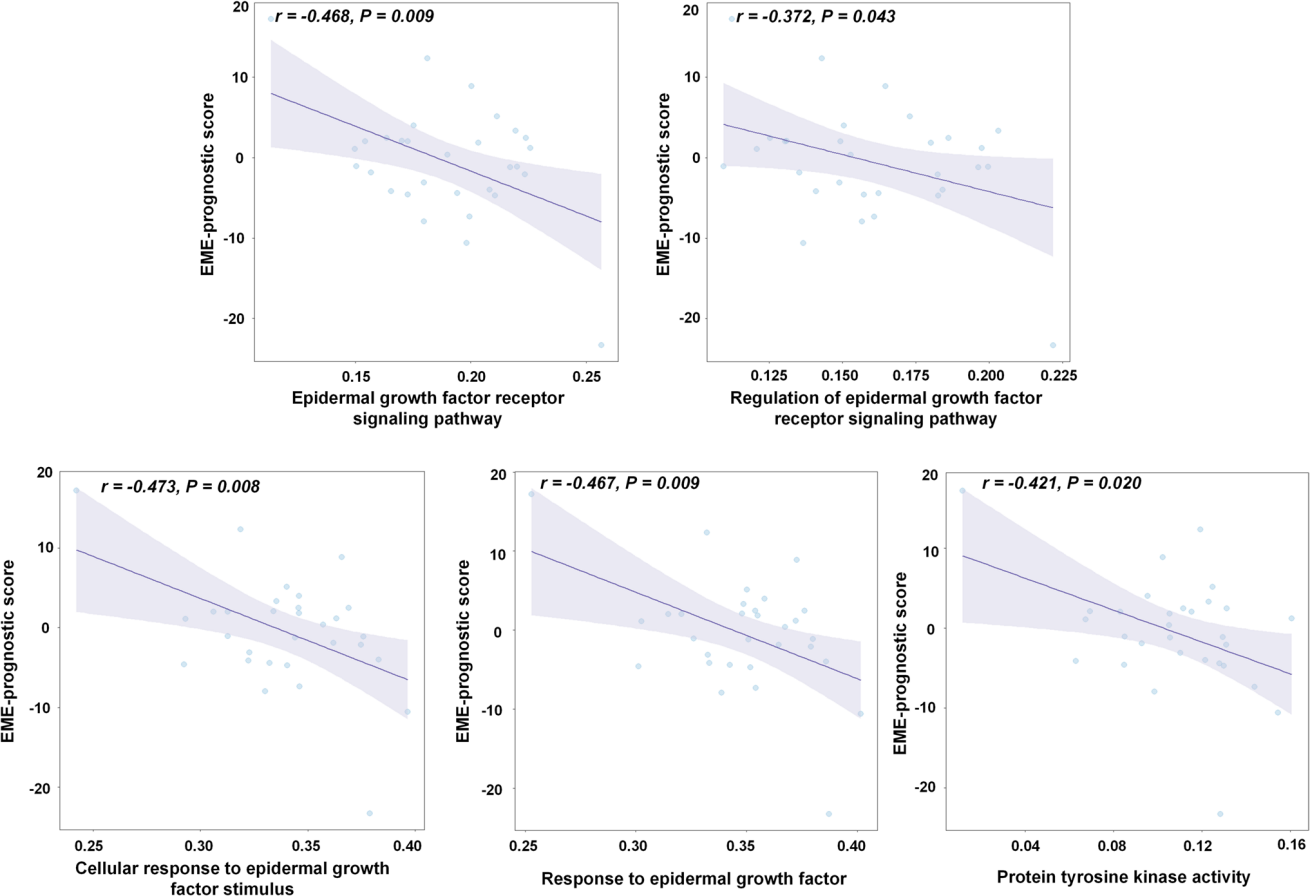
Correlation of the patient-level gene set variation analysis scores of top-ranked key pathways and the EME-prognostic score. The Pearson’s correlation coefficient (*r*) was presented with a false discovery rate-adjusted *P* < 0.05. EME, the deep learning model proposed in this study

In the TCGA-LUAD cohort, we found significant negative correlations between the EME-prognostic scores and five top-ranked biological pathways (EGFR signaling pathway, regulation of EGFR signaling pathway, cellular response to epidermal growth factor (EGF) stimulus, response to EGF, and protein tyrosine kinase activity, *P* < 0.05, see Fig. 6 and Supplementary Table S2). As mentioned above, the results indicated that as the EME-prognostic scores increased, patients were more unlikely to benefit from EGFR-TKI. These findings suggested that the prognosis of patients with NSCLC predicted by the EME was positively correlated with the expression of the pathways. Specifically, the results indicated that the survival prognosis predicted by the EME was positively correlated with the expression level of the EGFR signaling pathway and the regulation of the EGFR signaling pathway. As previously reported, patients with EGFR mutations or increased copy number of the EGFR gene are more likely to benefit from EGFR-TKI therapies and potentially show better survival outcomes [40, 41], suggesting that the EME model could potentially reveal the clinical outcome of patients with NSCLC which are regulated by the underlying biological mechanism of EGFR. Moreover, the results showed that the prognosis of patients with NSCLC predicted by the EME was significantly correlated with the pathways of cellular response to EGF stimulus and response to EGF and protein tyrosine kinase activity, which are associated with EGFR binding and kinase activity activation. This finding is consistent with previous reports that EGF binding activates the EGFR tyrosine kinase activity [42, 43].

## Discussion

In this study, we constructed and validated a deep learning system called EME to detect EGFR mutation status in patients with NSCLC, predict the prognosis of EGFR-mutant NSCLC patients receiving EGFR-TKI therapies, and reveal the biological correlation of the EME-prognostic score. The EME system showed competitive performance in predicting EGFR-mutant status and evaluating EGFR-TKI therapy prognosis. In addition, this study demonstrated the biological pathway correlation between the EME score and EGFR regulation in patients with NSCLC, indicating that the EME system is an efficient non-invasive approach for advancing individualized clinical decision-making for patients with NSCLC.

Previous studies have indicated that the proportion of patients with NSCLC with EGFR mutations has reached 10% in the USA and 40% in East Asia [44, 45]. Currently, the determination of EGFR mutation status relies on invasive testing of tumor tissue, which entails multiple manual interventions and is accompanied by significant spatial and temporal intratumoral heterogeneity [4]. Therefore, developing non-invasive and assessable radiological assessment for EGFR mutation detection and identifying patients who are likely to benefit from EGFR-TKI are crucial for clinical decision-making.

Compared with results from previous studies, the EME system achieved competitive accuracy in predicting EGFR-mutant status [8, 15]. Wang et al. proposed a deep learning model to predict the EGFR mutation status in lung adenocarcinoma using CT, and their model reached an AUC of 0.810 in the test dataset [15]. Recently, a new CT-based deep learning model was proposed to predict the EGFR mutation status and reached an AUC of 0.813 in the test dataset [8]. According to the results presented here, the EME model achieved an AUC of 0.907 (95% CI 0.840–0.926), which is superior to that of the previous models.

Due to individual differences, it is difficult to accurately identify patients who can benefit from EGFR-TKI in clinical practice. Even patients with detected EGFR mutations may not benefit from EGFR-TKI [5–7]. Previous studies have indicated the ability of deep learning models to evaluate the survival prognosis in EGFR-mutant NSCLC patients receiving EGFR-TKI therapies [8, 12, 13]. The EME-prognostic model proposed in this study further demonstrated its value in separating patients receiving EGFR-TKI treatment into high- and low-risk prognostic groups.

Furthermore, the majority of models proposed in previous studies were constrained to accomplish only one objective, namely predicting EGFR mutation status [15] or evaluating the prognosis of patients receiving EGFR-TKI therapies [16]. The EME model proposed in this study can not only detect EGFR mutation status, but it can also predict EGFR-TKI prognosis in patients with NSCLC. This capability facilitates early treatment initiation, thereby enhancing the potential for improved therapeutic outcomes. Moreover, since deep learning is a “black box,” the mechanisms involved are difficult to understand. Whereas previous studies have not further explained the internal mechanism of the reported deep learning models [15, 16], this study interprets it from heat maps and scatter distributions and identifies the relevant biological pathways, which improves the interpretability of the model from a biological point of view.

To further clarify the value of CT images in this context, we constructed radiomics models for comparison and fusion. In predicting EGFR mutation status, the radiomics results (Table 2) demonstrated good performance, which confirmed that medical images contain substantial information for detecting EFGR mutation-derived variations [46, 47]. Therefore, a fusion model containing EME and radiomics models was constructed. As listed in Table 2, the fusion model achieved the best performance. However, no statistically significant difference was observed between the EME and fusion models in the test dataset, indicating that the radiomics features were not significant for the fusion model in terms of accuracy improvement, which is consistent with previous works [48–50]. Furthermore, in contrast to radiomics models, the EME model does not necessitate the laborious task of manually outlining ROIs, which allows radiologists to be freed from the burdensome workload associated with segmentation, thereby increasing the acceptability of the EME model.

In terms of prognosis evaluation, radiomics features were used to construct a radiomics-prognostic model; however, the results on survival prognosis of the radiomics and fusion model were inferior (C-index < 0.600 on the test dataset), which is consistent with the findings of previous comparative studies [51, 52]. In contrast, the proposed method, in addition to detecting EGFR mutation status, enables the EME model to identify patients who are more likely to derive benefits from EGFR-TKI. This capability facilitates early treatment initiation, thereby enhancing the potential for improved therapeutic outcomes.

Recently, GRAD-CAM and t-SNE have been widely used to reveal hidden features of deep learning models [36, 53]. In this study, the GRAD-CAM activation maps demonstrated that our model primarily directed attention toward the intratumoral and peritumoral regions. This observation aligns with the findings of previous studies, which have concluded that activation maps can assist physicians in identifying high-risk lung areas in patients, thereby facilitating early intervention and enabling adjustments to treatment plans. In addition, the t-SNE analysis revealed a strong correlation between the EME features and EGFR-mutant status, indicating that the EME features correctly detect the distinct features on CT images between patients with EGFR-mutant and EGFR wild-type NSCLC. Moreover, according to the EME-prognostic model features in EGFR-mutant NSCLC patients, the differences between the patient subgroups with different prognostic risks in Fig. 5 revealed the potential feature divergence of patients with distinct responses to EGFR-TKI therapies, which aids in providing better individualized clinical treatment for patients with NSCLC. Moreover, our study demonstrated that the EME-prognostic score was significantly associated with biological pathways linked to the EGFR mutation and efficacy of EGFR-TKI, which could help clinicians better understand the prognostic information on radiological images that are regulated by these underlying biological pathways.

This study had some limitations. First, we only included Asian patients to develop and validate the EME model, and future research should be based on more ethnicities, in order to improve the universality of the deep learning model. Moreover, EGFR mutation subtypes, such as exon 19 deletions and L858R mutations [25], may be associated with patient prognosis; therefore, studies on different subtypes of EGFR mutations should further investigate this. Second, histopathological images may contain more information and should be considered in further studies to comprehensively analyze the image characteristics of EGFR-mutant NSCLC patients. Next, owing to the correlation between the clinical features and survival prognosis of patients with NSCLC [54], a fusion model containing clinical features should be considered. While we have uncovered the correlation between the EME and biological pathways, it is imperative to conduct in vitro or in vivo experiments to provide further evidence and substantiate these biological associations.

In conclusion, our study proposed a non-invasive and biologically interpretable method to detect the EGFR mutation status in patients with NSCLC and predict the prognosis of patients receiving EGFR-TKI based on pre-therapy CT images. The EME deep learning system can facilitate more individualized clinical decision-making for patients with NSCLC.

### Supplementary Information

Below is the link to the electronic supplementary material.

Supplementary file1 (DOCX 1.11 MB)

## Data Availability

The corresponding authors confirms that they had full access to all the study data and had the final responsibility for the decision to submit for publication.

## Abbreviations

AIC: Akaike information criterion
AUC: Area under the curve
C-index: Harrell’s concordance index
CI: Confidence interval
CT: Computed tomography
EGF: Epidermal growth factor
EGFR: Epidermal growth factor receptor
EME: The deep learning model proposed in this study
Grad-CAM: Gradient-weighted class activation maps
GSVA: Gene set variation analysis
HR: Hazard ratio
LUAD: Lung adenocarcinoma
NSCLC: Non-small cell lung cancer
PFS: Progression-free survival
SHAP: SHapley Additive exPlanation values
TCGA: The Cancer Genome Atlas
TKI: Tyrosine kinase inhibitors
t-SNE: T-distributed Stochastic Neighbor Embedding

## Declarations

### Disclaimer

The funders had no role in the study design, data collection and analysis, decision to publish, or preparation of the manuscript.
