# Supplementary material for: EfficientNet-Based System for Detecting EGFR-Mutant Status and Predicting Prognosis of Tyrosine Kinase Inhibitors in Patients with NSCLC
Source: J Imaging Inform Med. 2024 Feb 15;37(3):1086–99. doi: 10.1007/s10278-024-01022-z (PMC11169294; doi:10.1007/s10278-024-01022-z)
Supplement: Supplementary file 1 — Supplementary file1 (DOCX 1.11 MB) [file 10278_2024_1022_MOESM1_ESM.docx]

**Supplementary Material**

***Supplementary A: Inclusion and exclusion criteria of TCGA-LUAD dataset.***

The inclusion criteria were: (1) patients diagnosed with LUAD, (2) patients who underwent CT examination before treatment, and (3) patients with RNA sequencing records. Patients without any of the aforementioned records were excluded.

***Supplementary B: Details of EGFR testing workflow.***

To identify EGFR mutant status, tumor specimens were obtained by surgical resection or biopsy. EGFR mutations were detected on four tyrosine kinase domains (exons 18-21), which are most common in lung cancer patients. Mutation status was determined using next generation sequencing or the amplification refractory mutation system with human EGFR gene mutations detection kits. If any exon mutation was detected, the patient was identified as EGFR mutant-type; otherwise, the patient was identified as EGFR-wild type.

***Supplementary C: Details of CT acquisition and reconstruction parameters.***

For the CT protocols of patients with NSCLC enrolled in this study: patients from Shengjing Hospital of China Medical University were imaged with a CT slice of 1-mm thickness on a GE Revolution 256 scanner (GE Medical Systems, Waukesha, USA), or imaged with a CT slice of 5-mm thickness on a NeuViz 128 scanner (Neusoft, Shenyang, China) or on a NeuViz 64 scanner (Neusoft, Shenyang, China). Patients from the other participant institution were underwent contrast-enhanced/non-enhanced chest CT using SIMENS SOMATOM Definition Flash 64 row dual-source CT machine.

The patient took a supine position, raised his/her arms, and lung was scanned at the end of inhalation. Parameters: Tube voltage: 100 kV or 140 kV, tube current: Care Dose 4D, scanning layer thickness: 2 mm, reconstructed layer thickness: 2 mm, reconstructed layer spacing: 2 mm, matrix: 512 × 512, FOV: 350 mm × 350 mm. The enhanced scan was performed by a double-barrel high-pressure syringe to inject 70 to 90 ml of the non-ionic contrast agent iopromide intravenously into the cubital vein. The injection speed is 2.5 to 3.0 ml/s, and arterial phase images are obtained after 30 to 40 s of injection.

***Supplementary D: Details of the development of EME.***

The EME model was based on the cutting-edge EfficientNetV2-L model with the top layers replaced by a global average pooling layer and a dropout layer pre-trained on ImageNet. Then, a fully connected layer was added to fit the EGFR mutation status. Details of the development of EME were as follows: the batch size was set to 16, and the Adam optimizer was used. Additionally, the ReduceLROnPlateau function from Keras was used to adjust the learning rate with the following parameters: monitor=val_accuracy, factors=0.3, patience=2. Additionally, the EME model was developed using TensorFlow (version 2.8.0) on an NVIDIA GeForce RTX 3090 GPU.

***Supplementary E: Detailed description of the radiomics features.***

As we described in the main text, two experienced radiologists with over 5 years of experience delineated the boundary of the primary tumor of each NSCLC patient on CT scans. To comply with *The Image Biomarker Standardization Initiative* and enhance reproducibility, each CT image was normalized to [-1, 1] before feature extraction. For each patient, 944 radiomics features were extracted using Pyradiomics (Version 3.0.1) ^1^. The radiomics features consists of 14 shape features, 18 statistical features, and 912 textural features. The textural features consisted of 24 Gray Level Co-occurrence Matrix (GLCM) features, 16 Gray Level Run Length Matrix (GLRLM) features, 16 Gray Level Size Zone Matrix (GLSZM) features, 14 Gray Level Dependence Matrix (GLDM) features, and 5 Neighborhood Gray Tone Difference Matrix (NGTDM) features. Moreover, Wavelet and and Local Binary Pattern (LBP) were applied to yield corresponding derived images. When using Wavelet as the filters, L and H were set to the low-pass and the high-pass filtering, the wavelet decompositions of volume can be labeled as Wavelet_LHH, Wavelet_LHL, Wavelet_LLH, Wavelet_LLL, Wavelet_HHH, Wavelet_HHL, Wavelet_HLH and Wavelet_HLL.

All features were listed as follows:

1. Shape features: Elongation, Flatness, Least Axis Length, Major Axis Length, Maximum 2D Diameter Column, Maximum 2D Diameter Row, Maximum 2D Diameter Slice, Maximum 3D Diameter, Mesh Volume, Minor Axis Length, Sphericity, Surface Area, Volume Ratio, Voxel Volume.
2. Statistical features: 10 Percentile, 90 Percentile, Energy, Entropy, Inter quartile Range, Kurtosis, Maximum, Mean Absolute Deviation, Mean, Median, Minimum, Range, Robust Mean Absolute Deviation, Root Mean Squared, Skewness, Total Energy, Uniformity, Variance.
3. Textural features:
4. Gray Level Co-occurrence Matrix (GLCM) features: Auto correlation, Cluster Prominence, Cluster Shade, Cluster Tendency, Contrast, Correlation, Difference Average, Difference Entropy, Difference Variance, Inverse Difference, Inverse Difference Moment, Inverse Difference Moment Normalized, Inverse Difference Normalized, Informational Measure of Correlation 1, Informational Measure of Correlation 2, Inverse Variance, Joint Average, Joint Energy, Joint Entropy, MCC, Maximum Probability, Sum Average, Sum Entropy, Sum Squares.
5. Gray Level Run Length Matrix (GLRLM) features: Gray Level Non Uniformity, Gray Level Non Uniformity Normalized, Gray Level Variance, High Gray Level Run Emphasis, Long Run Emphasis, Long Run High Gray Level Emphasis, Long Run Low Gray Level Emphasis, Low Gray Level Run Emphasis, Run Entropy, Run Length Non Uniformity, Run Length Non Uniformity Normalized, Run Percentage, Run Variance, Short Run Emphasis, Short Run High Gray Level Emphasis, Short Run Low Gray Level Emphasis.
6. Gray-level size zone matrix (GLSZM) features: Gray Level Non Uniformity, Gray Level Non Uniformity Normalized, Gray Level Variance, High Gray Level Zone Emphasis, Large Area Emphasis, Large Area High Gray Level Emphasis, Large Area Low Gray Level Emphasis, Low Gray Level Zone Emphasis, Size Zone Non Uniformity, Size Zone Non Uniformity Normalized, Small Area Emphasis, Small Area High Gray Level Emphasis, Small Area Low Gray Level Emphasis, Zone Entropy, Zone Percentage, Zone Variance.
7. Gray Level Dependence Matrix (GLDM) features: Dependence Entropy, Dependence Non Uniformity, Dependence Non Uniformity Normalized, Dependence Variance, Gray Level Non Uniformity, Gray Level Variance, High Gray Level Emphasis, Large Dependence Emphasis, Large Dependence High Gray Level Emphasis, Large Dependence Low Gray Level Emphasis, Low Gray Level Emphasis, Small Dependence Emphasis, Small Dependence High Gray Level Emphasis, Small Dependence Low Gray Level Emphasis.
8. Neighboring Gray Tone Difference Matrix (NGTDM) features: Busyness, Coarseness, Complexity, Contrast, Strength.

**References**

1. van Griethuysen JJM, Fedorov A, Parmar C, Hosny A, Aucoin N, Narayan V, et al. Computational Radiomics System to Decode the Radiographic Phenotype. Cancer Res. 2017 Nov 1;77(21):e104-e7.

***Supplementary F: The Radiomics model for detecting EGFR mutant status***

Rad score = (-1.016×original_shape_Elongation)+(-1.550×original_shape_Flatness)+(-1.364×original_shape_Sphericity)+(0.001×wavelet_LLH_firstorder_Maximum)+(0.004×wavelet_LLH_firstorder_Median)+(0.091×wavelet_LLH_firstorder_Skewness)+(-2.120×10^-9^×wavelet_LLH_glszm_LargeAreaHighGrayLevelEmphasis)+(-0.138×wavelet_LLH_gldm_DependenceVariance)+(0.126×wavelet_LHL_firstorder_Mean)+(-0.479×wavelet_LHH_firstorder_Mean)+(1.054×10^-5^×wavelet_LHH_glcm_ClusterProminence)+(7.463×wavelet_LHH_glszm_GrayLevelNonUniformityNormalized)+(-52.580×wavelet_LHH_glszm_LowGrayLevelZoneEmphasis)+(0.537×wavelet_LHH_glszm_ZoneEntropy)+(-3.67×10^-6^×wavelet_LHH_gldm_LargeDependenceHighGrayLevelEmphasis)+(-0.113×wavelet_HLL_firstorder_Mean)+(29.490×wavelet_HLL_glszm_SmallAreaLowGrayLevelEmphasis)+(-0.854×wavelet_HLH_firstorder_Skewness)+(-3.685×wavelet_HLH_glcm_Correlation)+(-1.268×10^-4^×wavelet_HLH_ngtdm_Complexity)+(-0.236×wavelet_HLH_ngtdm_Strength)+(0.054×wavelet_HHL_firstorder_Kurtosis)+(0.006×wavelet_HHL_glcm_ClusterShade)+(1.435×wavelet_HHL_glrlm_LongRunLowGrayLevelEmphasis)+(-0.174×wavelet_HHL_glszm_GrayLevelNonUniformityNormalized)+(-3.335×wavelet_HHL_glszm_SmallAreaEmphasis)+(0.037×wavelet_HHL_gldm_DependenceVariance)+(-15.610×wavelet_HHH_glcm_InverseVariance)+(8.705×wavelet_HHH_glszm_GrayLevelNonUniformityNormalized)+(-33.200×wavelet_HHH_ngtdm_Contrast)+(111.400×wavelet_HHH_gldm_SmallDependenceLowGrayLevelEmphasis)+(3.257×wavelet_LLL_ngtdm_Busyness)+(-0.387×lbp_2D_firstorder_InterquartileRange)+(-5.914×lbp_2D_glszm_SmallAreaEmphasis)+(0.060×lbp_2D_gldm_DependenceVariance)+3.544

***Supplementary G: The fusion model for detecting EGFR mutant status***

Fusion score = (-12.940×EME score)+(-0.152×wavelet_LLH_firstorder_Entropy)+(0.001×wavelet_LLH_firstorder_Maximum)+(0.005×wavelet_LLH_firstorder_Median)+(2.172×wavelet_LLH_glszm_ZoneEntropy)+(4.180×wavelet_LHH_glcm_Imc2)+(-0.370×wavelet_LHH_glszm_ZoneEntropy)+(-1.806×10^-5^×wavelet_LHH_gldm_LargeDependenceHighGrayLevelEmphasis)+(-0.131×wavelet_HLH_glcm_Correlation)+(11.160×wavelet_HHL_glcm_Correlation)+(4.612×wavelet_HHL_glrlm_ShortRunLowGrayLevelEmphasis)+(0.005×wavelet_HHH_glszm_GrayLevelVariance)+(0.437×wavelet_LLL_glrlm_RunEntropy)+(-1.828×lbp_2D_glszm_SizeZoneNonUniformityNormalized)+(0.146×lbp_2D_gldm_DependenceVariance)-14.530

***Supplementary H: The EME-prognostic model.***

EME-prognostic score = (-988.135×Feature10)+(414.382×Feature12)+(-756.177×Feature27)+(-1045.720×Feature35)+(1280.397×Feature58)+(-702.288×Feature86)+(698.359×Feature91)+(-308.490×Feature98)+(-501.454×Feature114)+(-1077.977×Feature115)+(1442.934×Feature127)+(680.555×Feature128)+(1074.925×Feature135)+(-446.629×Feature141)+(1300.244×Feature149)+(310.632×Feature164)+(520.731×Feature176)+(-391.752×Feature203)+(-1120.056×Feature224)+(-876.473×Feature238)+(140.487×Feature250)+(1137.613×Feature269)+(-693.047×Feature298)+(-666.728×Feature326)+(-245.349×Feature354)+(818.585×Feature371)+(722.965×Feature431)+(-737.884×Feature442)+(451.997×Feature446)+(1006.928×Feature503)+(-545.536×Feature517)+(573.759×Feature611)+(-964.182×Feature633)+(1447.108×Feature640)+(1663.350×Feature702)+(574.018×Feature754)+(-1583.629×Feature799)+(193.714×Feature963)+(-771.038×Feature971)+(-1003.056×Feature979)+(-831.376×Feature1009)+(232.525×Feature1017)+(-1177.406×Feature1024)+(322.700×Feature1121)+(-501.870×Feature1125)+(588.711×Feature1212)+(211.860×Feature1217)+(-446.752×Feature1230)+(782.075×Feature1258)+28.241

***Supplementary Tables***

**Table S1.** Characteristics of the included patients from TCGA-LUAD.

|  | TCGA-LUAD dataset |
| --- | --- |
| N (%) | 30 (100%) |
| Age, N (%) |  |
| <65 | 9 (30.0%) |
| ≥65 | 21 (70.0%) |
| Sex, N (%) |  |
| Male | 8 (26.7%) |
| Female | 22 (73.3%) |
| Race Category, N (%) |  |
| White | 27 (90.0%) |
| Black or African American | 3 (10.0%) |
| Progression Free Status |  |
| Censored, N (%) | 9 (30.0%) |
| Progression, N (%) | 21 (70.0%) |

Note: TCGA-LUAD, The Cancer Genome Atlas-Lung Adenocarcinoma.

**Table S2.** Top-ranked biological pathways correlated with the EME-prognostic score. The top-ranked biological pathways were obtained using *clusterProfiler* package by querying the Gene Ontology Biologic Process. Biological pathways with an FDR-adjusted P <0.05 were considered to be statistically significant in the enrichment analysis.

| **Pathway** | **P value** | **Genes** | **Dataset** |
| --- | --- | --- | --- |
| Epidermal growth factor receptor signaling pathway | 0.009 | ABL1, ADAM17, ADORA1, ADRA2A, AFAP1L2, AGR2, AGT, AKT1, AREG, ARF4, BCAR1, BCAR3, BRAF, BTC, CBL, CBLB, CBLC, CCDC88A, CDH13, CEACAM1, CHMP6, DAB2IP, DGKD, DUSP3, EFEMP1, EGF, EGFR, EREG, ERRFI1, FAM83A, FAM83B, FASLG, FBXW7, FER, GAB1, GPRC5A, GRB2, GRB7, HAP1, HBEGF, HIP1, HIP1R, IFI6, IQGAP1, ITGA1, KIF16B, MMP9, MVP, NCF1, NCK2, NEU3, NUP62, PDE6G, PDPK1, PIGR, PIK3C2A, PIK3CA, PLAUR, PLCE1, PLCG1, PSEN1, PTK2, PTK2B, PTK6, PTPN11, PTPN12, PTPN2, PTPN3, PTPRJ, RAB7A, RASSF2, REPS2, RHBDF1, RHBDF2, RNF115, RNF126, RPS6KA5, SHC1, SHC3, SHKBP1, SNX5, SNX6, SOCS4, SOCS5, SOS1, SOX9, SRC, TGFA, TGFB1, TSG101, VIL1, VPS25, WDR54, ZFYVE28, ZGPAT | Gene Ontology Biological Processes |
| Regulation of epidermal growth factor receptor signaling pathway | 0.043 | ADAM17, ADORA1, ADRA2A, AFAP1L2, AGR2, AGT, AREG, BCAR3, BTC, CBL, CBLB, CBLC, CCDC88A, CDH13, CEACAM1, CHMP6, DAB2IP, DGKD, DUSP3, EGF, EGFR, EREG, ERRFI1, FASLG, FBXW7, FER, GPRC5A, HAP1, HBEGF, HIP1, HIP1R, IFI6, ITGA1, MMP9, MVP, NCF1, NEU3, NUP62, PDE6G, PLAUR, PSEN1, PTK6, PTPN12, PTPN2, PTPN3, PTPRJ, RAB7A, RHBDF1, RHBDF2, RNF115, RNF126, SHKBP1, SNX5, SNX6, SOCS4, SOCS5, SOS1, TGFA, TSG101, VPS25, WDR54, ZFYVE28, ZGPAT | Gene Ontology Biological Processes |
| Cellular response to epidermal growth factor stimulus | 0.008 | AKT1, BAG4, BAIAP2, BECN1, CAD, CBL, CFLAR, COL1A1, DAB2IP, DUSP22, DUSP3, EEF1A1, EGFR, ERBB2, ERBB4, ERRFI1, FOXC1, GSTP1, ID1, INPP5K, IQGAP1, MCM7, MED1, NCL, PDE8A, PDPK1, PLCG1, PPP1R9B, PTPN11, PTPN12, SNAI2, SNX6, SOX9, STAT5B, SYAP1, VIL1, ZFP36, ZFP36L1, ZFP36L2 | Gene Ontology Biological Processes |
| Response to epidermal growth factor | 0.009 | AKT1, BAG4, BAIAP2, BECN1, CAD, CBL, CFLAR, COL1A1, DAB2IP, DUSP22, DUSP3, EEF1A1, EGFR, ERBB2, ERBB4, ERRFI1, FOXC1, GSTP1, ID1, INPP5K, IQGAP1, MAPK1, MAPK3, MCM7, MED1, NCL, PDE8A, PDPK1, PLCG1, PPP1R9B, PTPN11, PTPN12, SNAI2, SNX6, SOX9, STAT5B, SYAP1, TPR, VIL1, ZFP36, ZFP36L1, ZFP36L2 | Gene Ontology Biological Processes |
| Protein tyrosine kinase activity | 0.020 | AATK, ABL1, ABL2, AXL, BAZ1B, BCR, BLK, BMX, BTK, CAMKK2, CLK1, CLK2, CLK3, CLK4, CRIM1, CSF1R, CSK, DDR1, DDR2, DSTYK, DYRK1A, DYRK1B, DYRK2, DYRK3, DYRK4, EFEMP1, EFNA3, EFNA4, EFNB3, EGFR, EIF2AK2, EPHA10, EPHA1, EPHA2, EPHA3, EPHA4, EPHB1, EPHB2, EPHB3, EPHB4, EPHB6, ERBB2, ERBB3, ERBB4, FER, FES, FGFR1, FGFR2, FGFR3, FGFR4, FGFRL1, FGR, FLT1, FLT3, FLT4, FRK, FYN, HCK, HIPK1, HIPK2, HIPK3, IGF1R, IGF2R, INSR, ITK, JAK1, JAK2, JAK3, KDR, KIT, LCK, LTK, LYN, MAP2K1, MAP2K2, MAP2K3, MAP2K4, MAP2K5, MAP2K6, MAP2K7, MATK, MELK, MERTK, MET, MST1R, NEK1, NRP1, NRP2, NTRK1, NTRK2, NTRK3, PBK, PDGFRA, PDGFRB, PDGFRL, PKDCC, PRKCD, PTK2, PTK2B, PTK6, RET, RIPK2, ROR2, ROS1, SCYL1, PEAK1, SRC, STK16, STYK1, SYK, TEC, TEK, TESK1, TESK2, TIE1, TNK1, TNK2, TTK, TTN, TXK, TYK2, TYRO3, WEE1, YES1, ZAP70 | Gene Ontology Biological Processes |

Note: EME,the deep learning model proposed in this study; FDR, Biological pathways with a false discovery rate

***Supplementary Figures***

**
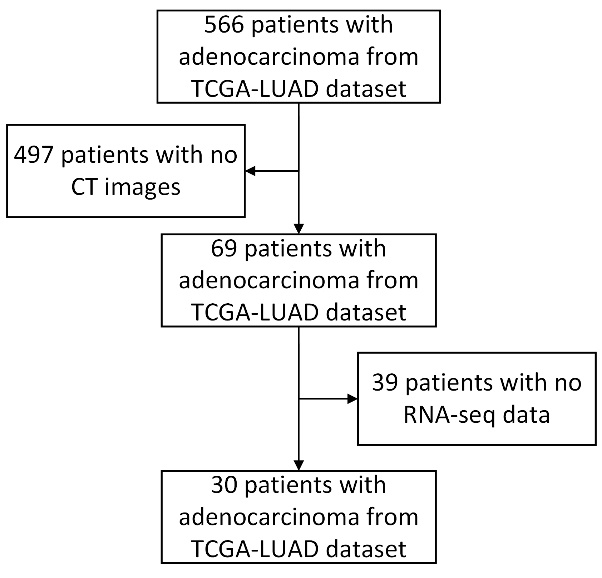
**

**Figure S1.** Patient of TCGA-LUAD cohort enrollment. TCGA-LUAD, The Cancer Genome Atlas-Lung Adenocarcinoma.


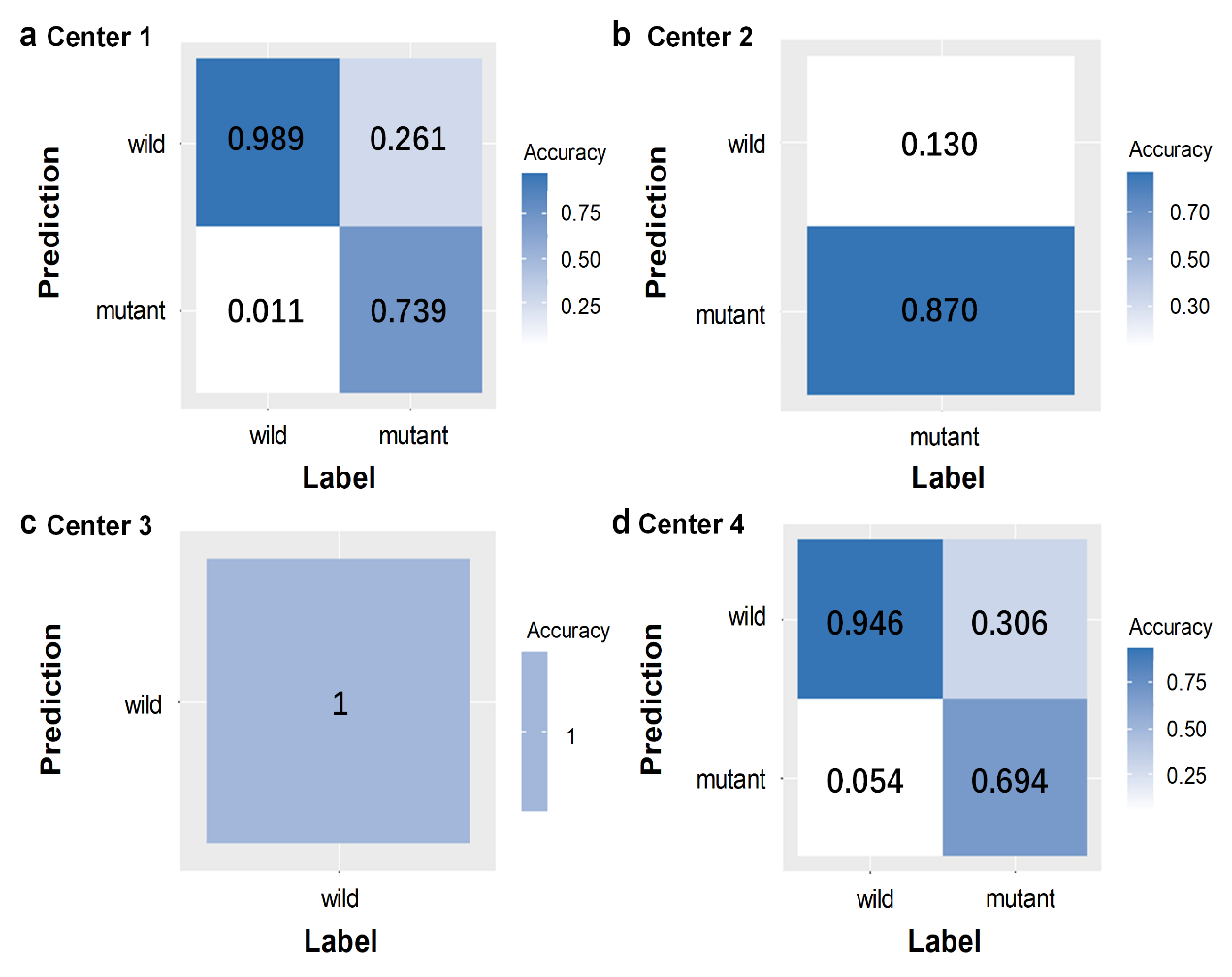


**Figure S2.** Confusion matrices in (a) center 1; (b) center 2; (c) center 3; (d) center 4. * Because Center 3 is only composed of EGFR wild-type patients, a 100% accuracy rate has been achieved.


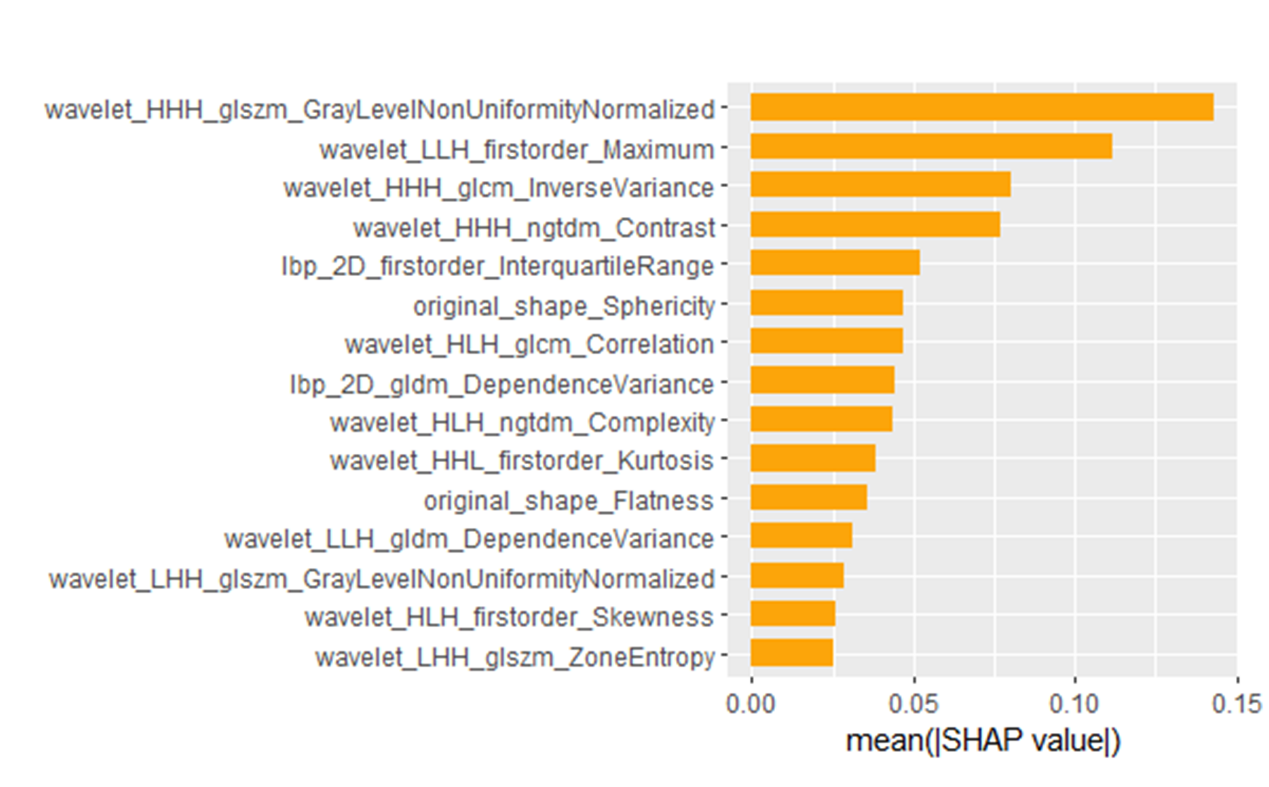


**Figure S3.** The contribution and importance (SHAP values) of top 15 radiomics features in radiomics model for predicting EGFR mutation status.

Note: SHAP, SHapley Additive exPlanation (SHAP) values.


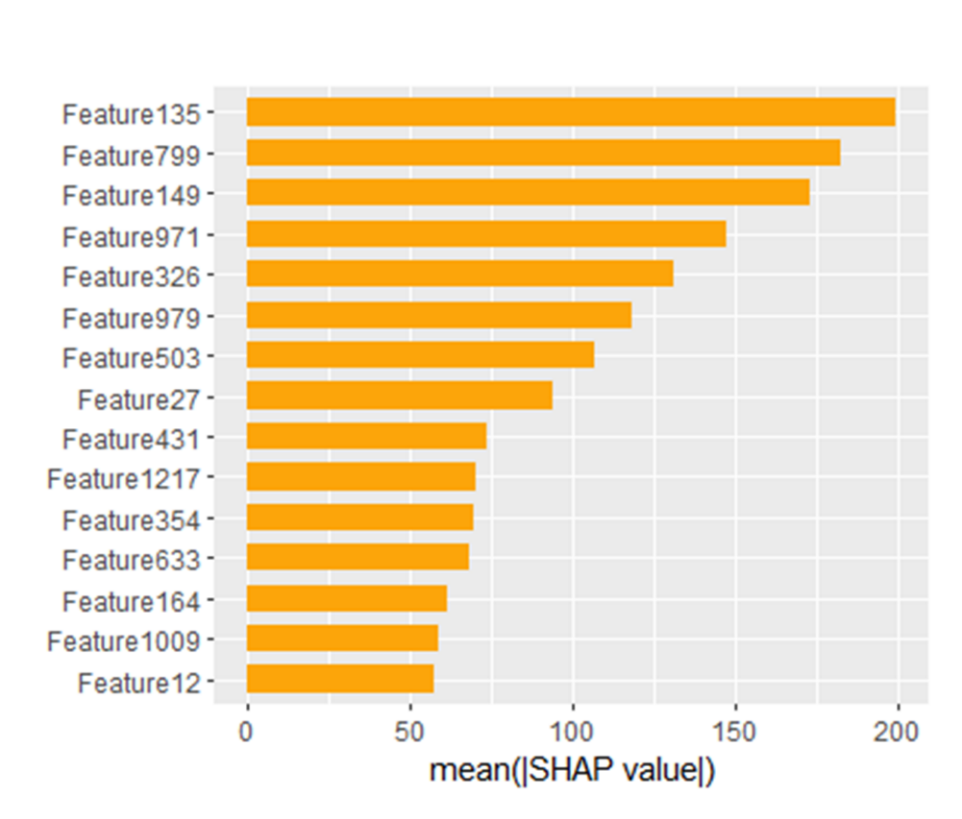


**Figure S4.** The contribution and importance (SHAP values) of top 15 EME features in EME-prognostic model.

Note: SHAP, SHapley Additive exPlanation (SHAP) values.
